# Supplementary material for: Multidomain architecture of estrogen receptor reveals interfacial cross-talk between its DNA-binding and ligand-binding domains
Source: Nat Commun. 2018 Aug 30;9:3520. doi: 10.1038/s41467-018-06034-2 (PMC6117352; doi:10.1038/s41467-018-06034-2)
Supplement: Supplementary file 1 — Supplementary Information [file 41467_2018_6034_MOESM1_ESM.pdf]

Supplementary Information

Multidomain architecture of estrogen receptor reveals interfacial cross-talk between its DNA-binding and ligand-binding domains

W. Huang et al., 2018

Supplementary Figures

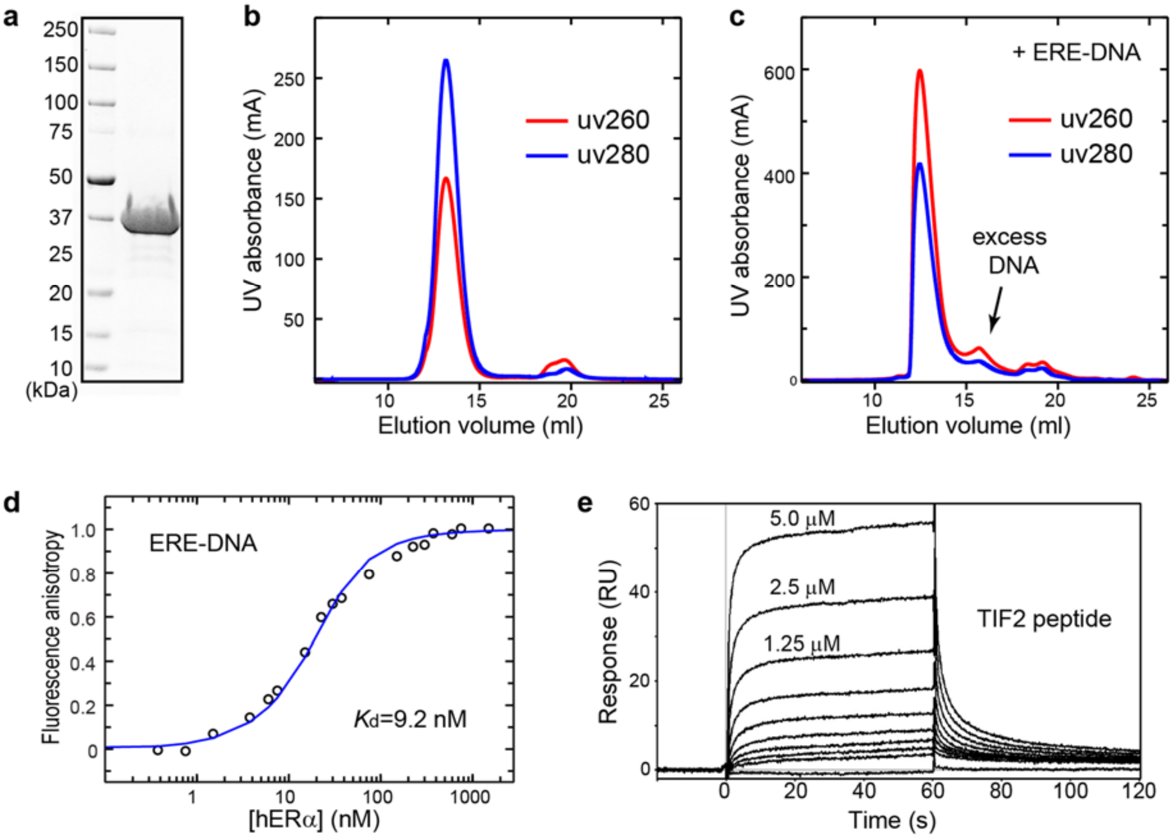

Supplementary Figure 1. Purification and characterization of recombinant hERα.

(**a**) Expression of hERα^{CDE}. Shown is the SDS-PAGE gel of recombinant hERα^{CDE} (E181-P552).

(**b**, **c**) Purification of hERα^{CDE}. Size exclusion chromatography (SEC) of hERα^{CDE} in the absence (B) or presence (C) of ERE-DNA. Shown are the absorbance at 260 nm (red) and 280 nm (blue) of hERα^{CDE} in the presence of estradiol and a coactivator TIF2 peptide.

(**d**) Binding to ERE-DNA of hERα^{CDE}. Fluorescence anisotropy was measured using a fluorescent dye, 6-Carboxyfluorescein (6-FAM) to label the 5' end of ERE-DNA, yielding an apparent binding affinity of $K_d = 9.2$ nM. Data of 2.8-μM hERα^{CDE} in the presence of E2 were used as a saturation level and free 20-nM DNA data as a baseline.

(**e**) Binding of a coactivator TIF2 peptide to hERα^{CDE}. The binding of the hERα to a coactivator TIF2 peptide (sequence KENALLRYLLDKDD) in the presence of E2 and ERE-DNA was measured by surface plasmon resonance (SPR). We hereafter refer to the hERα^{CDE} in the estradiol E2, ERE-DNA, and a coactivator TIF2 peptide as hERα^{complex}.

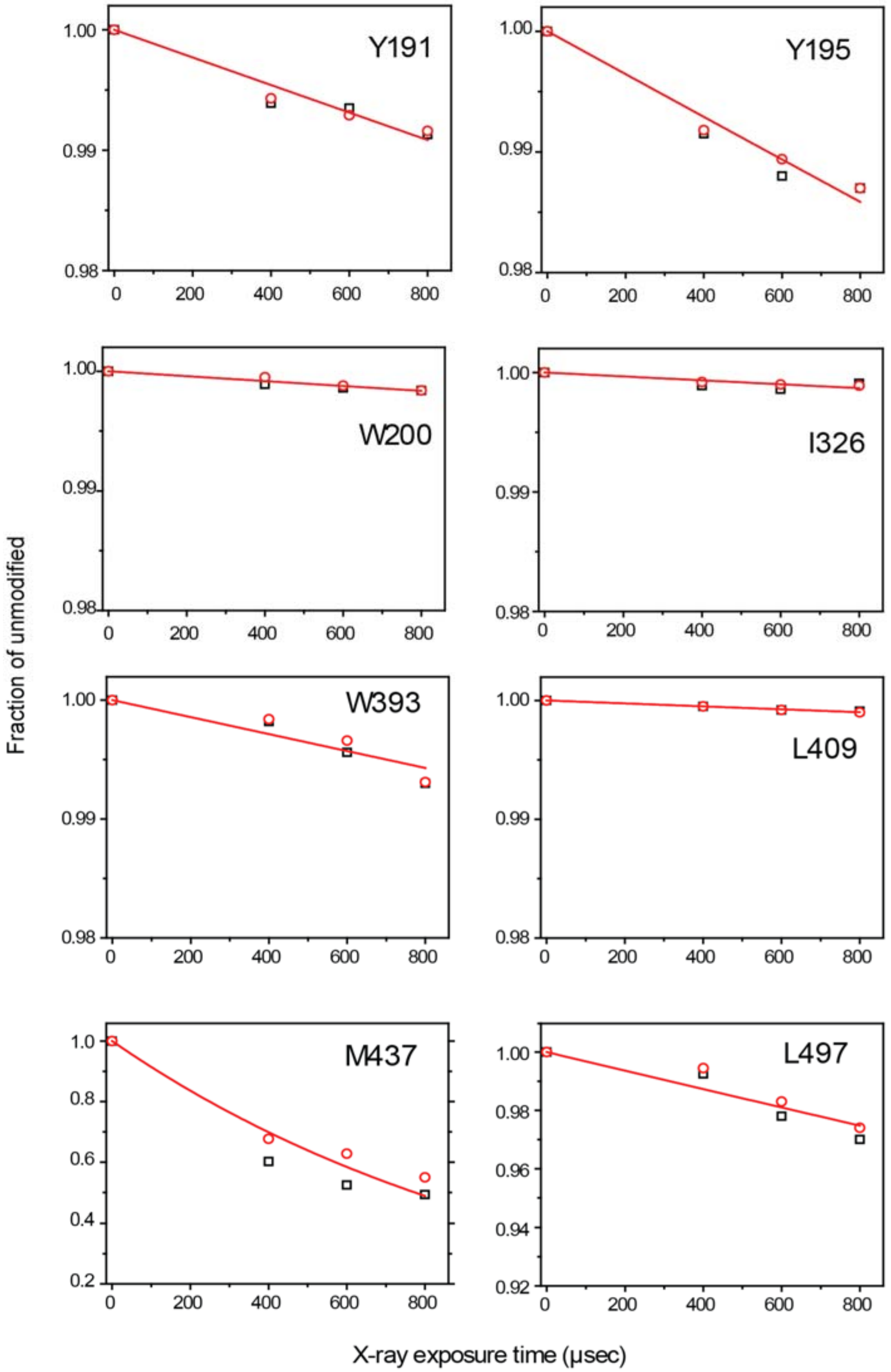

Supplementary Figure 2. Dose-response plots of footprinting as a function of X-ray exposure time. Red line is the least-squares fit of the averages of duplicate dose-dependent data (i.e., a normalized fraction of unmodified residues), each yielding a rate of footprinting.

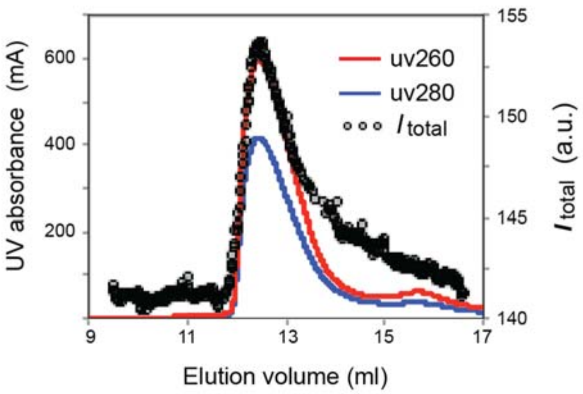

Supplementary Figure 3. Chromatography-coupled SAXS data acquisition of hERα. UV absorbance and total scattering intensity were shown along the Size exclusion chromatography (SEC) elution of hERα^{complex}. Total scattering intensity, I_{total}, was calculated by integrating all scattering signals over the entire q-range (up to 0.3 Å^{-1}). SEC-SAXS data at the elution peak have been deposited into SASBDB (access code SASDDU8; https://www.sasbdb.org/data/SASDDU8).

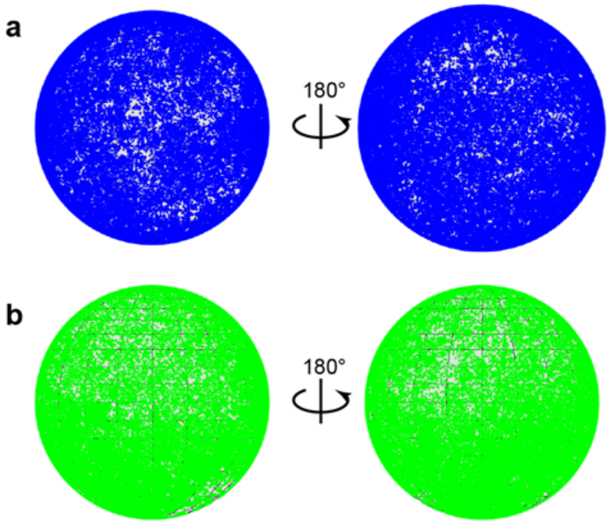

Supplementary Figure 4. Sampling of computational docking. Docked configurations (dot) were projected onto a unit globe sampled by coarse-grained simulations. (**a**) The projection of the center-of-mass of the DBD dimer on the surface of the LBD dimer. (**b**) The projection of the LBD dimer on the surface of the DBD dimer.

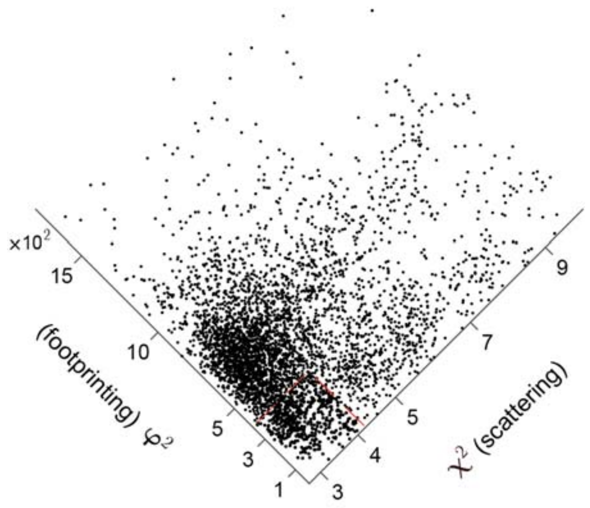

Supplementary Figure 5. Data integration by fitting docking results against experimental data. The fit of computationally generated conformations (dot) is simultaneously assessed against footprinting (φ^2) and scattering (χ^2). The region marked with the red lines is highlighted in the main text.

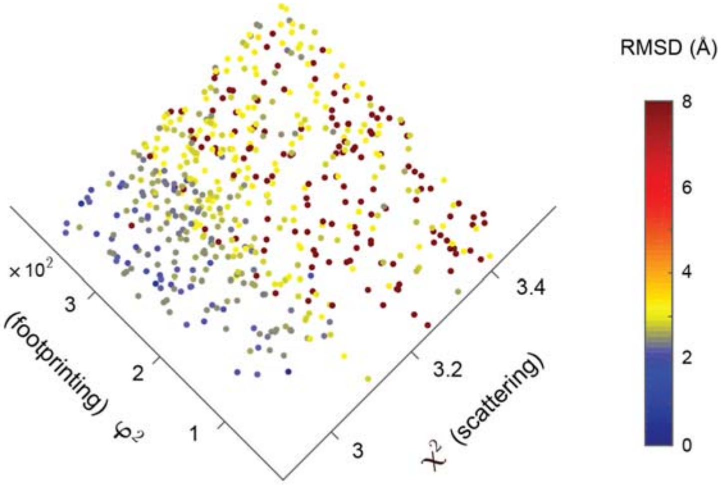

Supplementary Figure 6. Cα-RMSD of docking structure models from the best-fit structure. Both LBD and DBD were included, except the domain-connecting hinge.

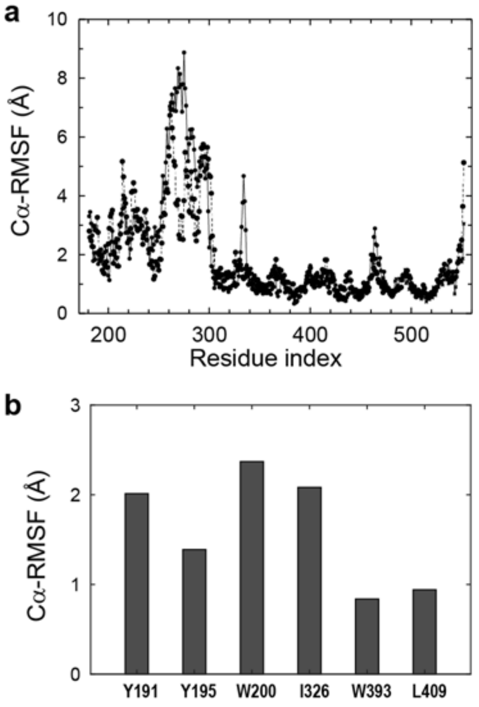

Supplementary Figure 7. Cα-RMS fluctuations (RMSF) from the hERα structure-ensemble. Residue RMSF values of both chains of the hERα homodimer (**a**) and for those footprinting detected interfacial residues (**b**).

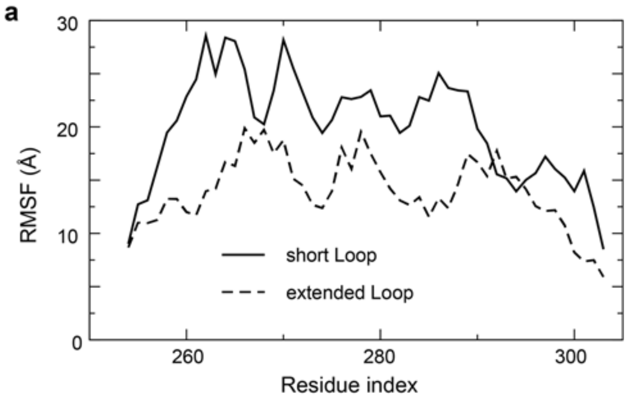

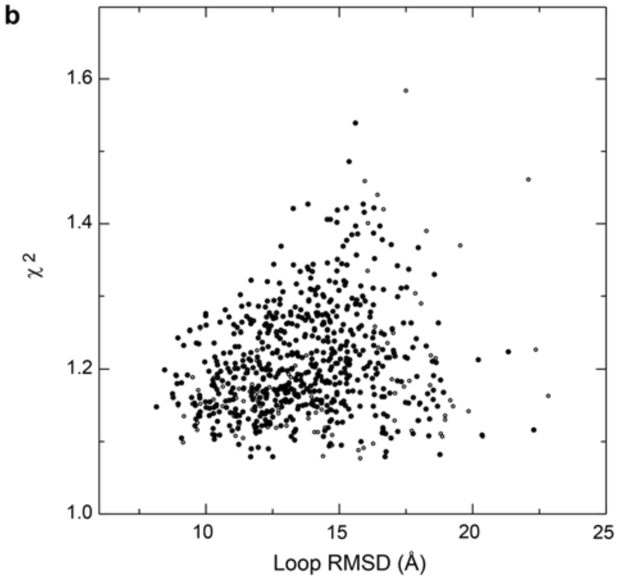

Supplementary Figure 8. Loop flexibility and its contribution to scattering fitting.

(**a**) Cα-atom RMS fluctuation (Cα-RMSF) of the DBD-LBD connecting loops (residues 254-303) for each peptide chain (short and extended).

(**b**) The goodness of fit χ^2 as a function of the loop's RMSD using its average structure as a reference. Scattering was calculated using the program *Crysol*. The loop structures were generated using the program *loopy* (http://honig.c2b2.columbia.edu/loopy/), where a set of 100 structures was used for each of hERα ensemble-structures (seven as shown in Fig. 2), yielding the value of χ^2=1.2 ± 0.1.

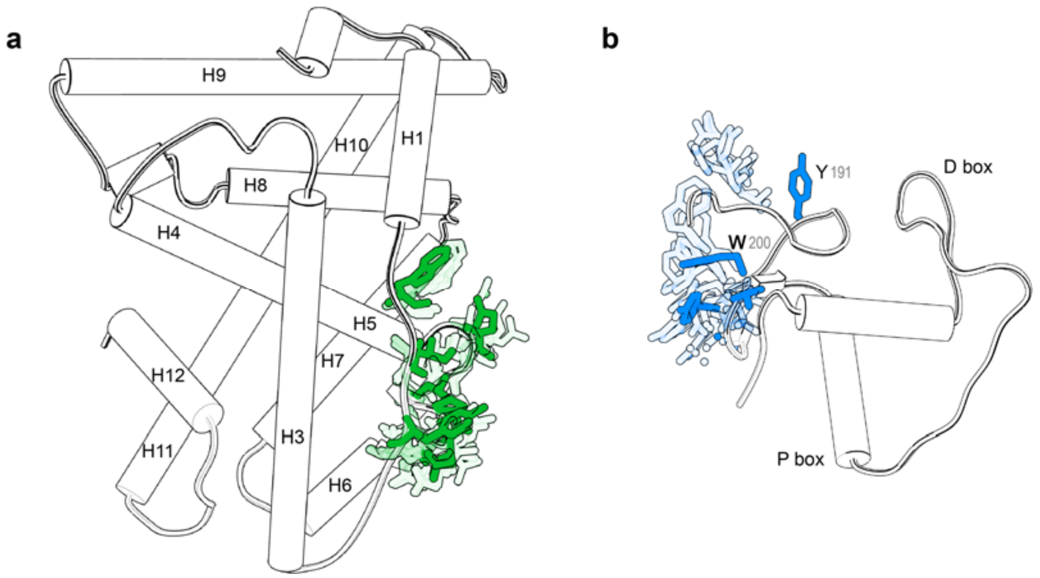

Supplementary Figure 9. Overlap of interfacial residues from individual domains and from the hERα ensemble-structures. Contact residues are colored on the LBD surface (**a**) and the DBD surface (**b**), where the side-chain conformations are shown in light (complex) and dark (crystal structures of individual domains) colors.

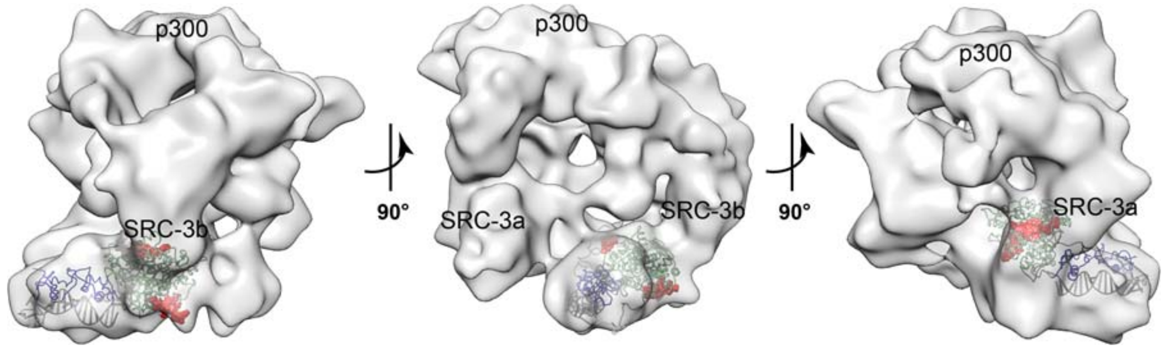

Supplementary Figure 10. Fitting of hERα ensemble structures into a 22-Å EM map. The ensemble-structures of hERα^{complex} were rigidly docked into the EM map (EMD-8832) of the full-length hERα in complex with coactivators (SRC-3a/SRC-3b/p300) was released and downloaded on September 20, 2017. The coactivator peptide (KENALLRYLLDKDD) used in our sample was highlighted in red, consistent with the positions of coactivators SRC-3a and SRC-3b as shown in the EM map.

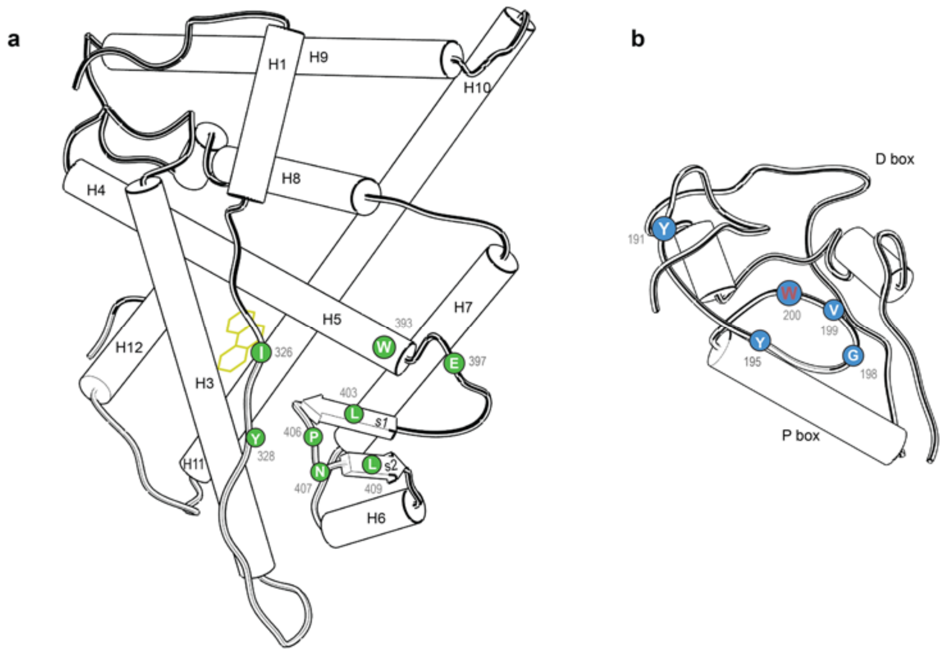

Supplementary Figure 11. Schematic drawing of hERα residues involved in the LBD-DBD interface.

(**a**) The LBD consists of 12 helices and two β-strands (s1 and s2 between helices H5 and H6). The interfacial residues are I326, Y328, W393, E397, L403, P406, N407, and L409. They form a shallow and nearly flat surface, which is distant from helix H12 (covering the ligand binding pocket) and coactivator binding sites (near helices H3 and H4). The bound ligand estradiol is depicted in yellow.

(**b**) The contact residues from the DBD side are Y191, Y195, G198, V199, and W200, before the first helix of the DBD.

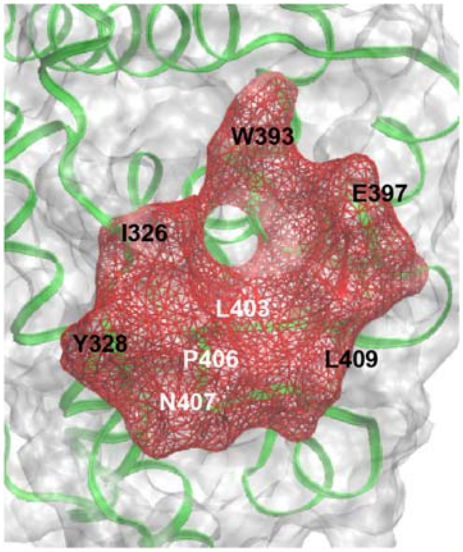

Supplementary Figure 12. The LBD surface involved in DBD-LBD interactions. A modest-sized pocket (red) consists of nine residues, I326, Y328, W393, E397, L403, P406, N407, and L409 on the molecular surface (gray) of the LBD (green).

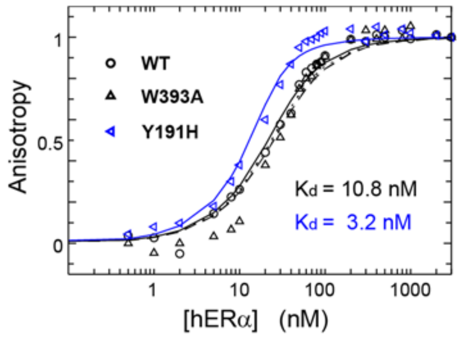

Supplementary Figure 13. Mutation Y191H increases DNA-binding. Y191H increases the receptor's DNA-binding affinity to K_d = 3.2 nM, compared to the WT binding affinity K_d = 9.2 nM. As a control, the W393A mutation has little change in its DNA-binding affinity K_d = 10.8 nM.

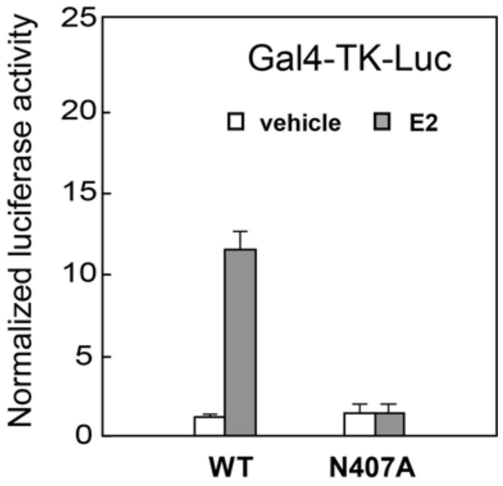

Supplementary Figure 14. Transient transfection reporter activity of the wild-type hERα and the mutant N407A from the Gal4-DBD/hERα-LBD fusion protein using a Gal4-TK-Luc reporter construct when treated with estradiol (E2).

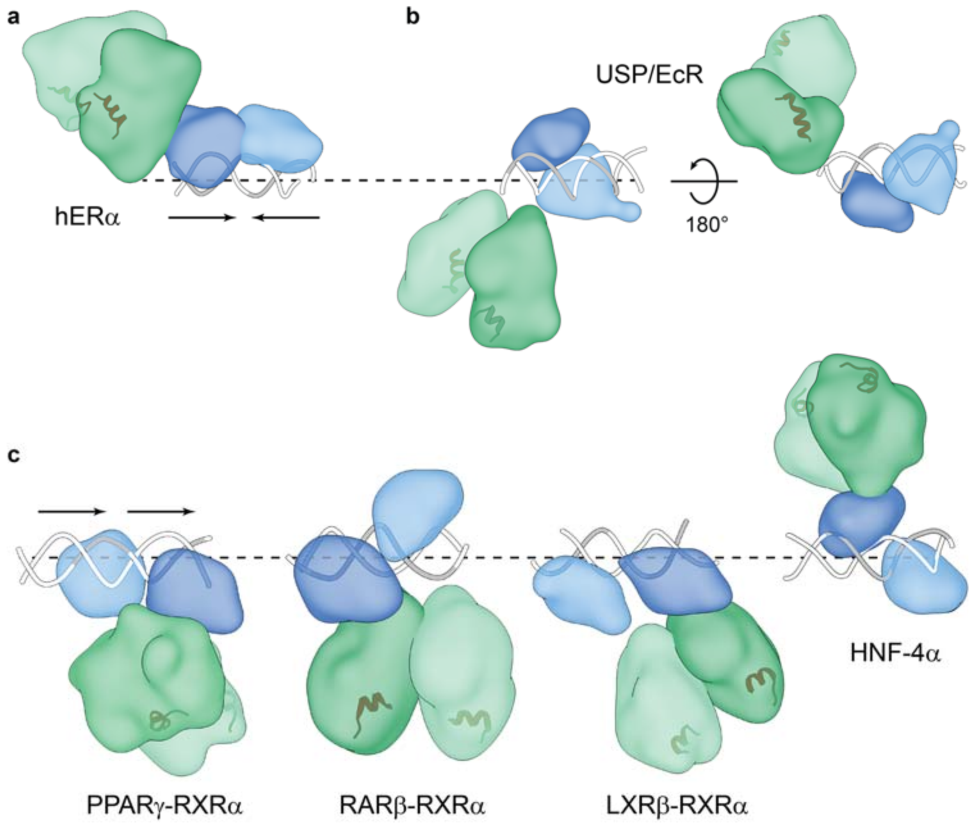

Supplementary Figure 15. Schematic representation of multidomain architectures of nuclear receptor complexes in complex with DNA of inverted repeat (IR) and direct repeat (DR).

(**a**) The hERα in complex with IR ERE-DNA (SASBDB entry SASDDU8; https://www.sasbdb.org/data/SASDDU8).

(**b**) IR-bound USP/EcR (PDB entry 4UMM). A rotated view is shown for comparison with hERα.

(**c**) DR-bound complexes of PPARγ-RXRα (PDB entry 3DZY), RARβ-RXRα (PDB entry 5UAN), RXRα-LXRβ (PDB entry 4NQA), and HNF-4α (PDB entry 4IQR). All structures are shown by aligning the DNA (the 5'-half of the first strand).

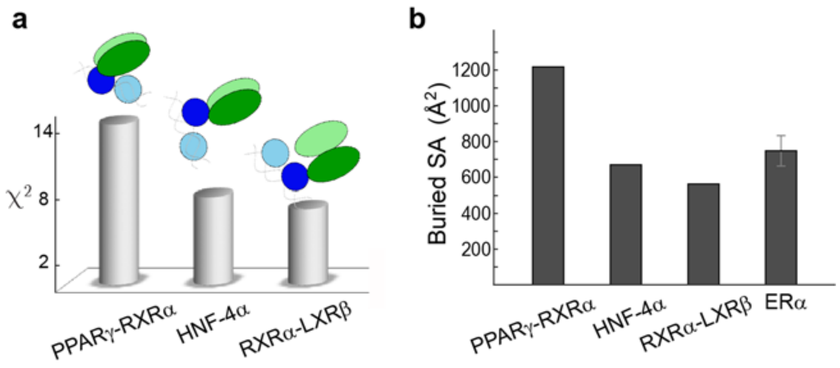

Supplementary Figure 16. Structural difference among multi-domain NRs.

(**a**) Large difference in overall architecture. Mismatch of domain-domain organizations of other NRs is demonstrated by their large differences with experimental SAXS data of hERα^{complex}. A structural model of hERα^{CDE} was built by threading of hERα^{CDE} sequence onto each NR structure to calculate its theoretical scattering profile. The resulting χ^2 values are 14.6, 8.0, and 6.9 for PPARγ-RXR, HNF-4α and LXRβ-RXRα, respectively. The domain-connecting hinge was built using the loop modeling package *loopy* (http://honig.c2b2.columbia.edu/loopy).

(**b**) Buried LBD surface. Buried solvent accessible surface areas (SA; s.d. from the structure-ensemble) are the difference of the LBD in the absence and presence of DBD. SA was calculated with a probe size of 1.4 Å using the software VMD.

Supplementary Tables

Supplementary Table 1. The footprinting rates (k_{FP}) and protection factors (logPF) of individual residues probed by footprinting. The twenty residues were divided into two groups: One is a control set of 14 residues that were used for a linear regression and the other includes six residues with large changes in logPF and SA values. SA values were calculated from the domain structures. The SA^{fit} values are the theoretical estimates inferred from the linear fit, where negative SA^{fit} values suggest that they are not solvent-exposed in the complex.

	Residue	k_{fp} (s^{-1})	logPF	SA (Å^2)	SA^{fit} (Å^2)
Control (14)	H196	123.30 ± 3.31	-2.59 ± 0.03	18.0	44.2
	H197	15.48 ± 0.87	-0.26 ± 0.06	4.5	13.4
	F208	5.94 ± 0.51	0.64 ± 0.09	0.1	1.6
	M220	575.30 ± 13.90	-3.33 ± 0.02	45.0	54.1
	M315	21.17 ± 0.98	-0.03 ± 0.05	2.3	10.4
	P325	4.29 ± 0.25	-1.46 ± 0.06	48.5	29.2
	M343	28.01 ± 1.23	-0.31 ± 0.04	6.7	14.1
	M357	66.64 ± 6.02	-1.18 ± 0.09	12.0	25.6
	L408	1.26 ± 0.05	1.25 ± 0.04	7.4	-6.6
	M427	118.51 ± 6.97	-1.75 ± 0.06	29.5	33.2
	M437	895.60 ± 74.28	-3.78 ± 0.08	69.6	59.9
	K492	4.87 ± 1.80	-0.79 ± 0.37	32.0	26.0
	L495	55.16 ± 2.74	-2.53 ± 0.05	50.3	43.4
	L497	30.21 ± 4.29	-1.92 ± 0.14	72.8	35.4
Prediction (6)	Y191	11.50 ± 0.53	0.04 ± 0.05	147.6	9.5
	Y195	17.80 ± 0.70	-0.39 ± 0.04	104.1	15.2
	W200	2.05 ± 0.11	2.14 ± 0.05	106.1	-18.3
	I326	1.64 ± 0.19	0.99 ± 0.12	77.7	-3.0
	W393	7.15 ± 0.76	0.89 ± 0.11	73.4	-1.8
	L409	1.24 ± 0.03	1.27 ± 0.02	45.9	-6.8

Supplementary Table 2. Primer sequences used for hERα constructs

Construct	Forward Primer	Reverse Primer
hERα^{CDE}	5'-GGTGAATTCGAGACTCGCTACTGTGC-3'	5'-ATTGGATCCTCAGGGCGCATGGATGG-3'
Y191H	5'-GTGCAGTGTGCAATGACCATGCTTCAGGCTACCATTATG-3'	5'-CATAATGGTAGCCTGAAGCATGGTCATTGCACACTGCAC-3'
I326A	5'-TTGGATGCTGAGCCCCCCGCACTCTATTCCGAGTATGAT-3'	5'-ATCATACTCGGAATAGAGTGCGGGGGGCTCAGCATCCAA-3'
Y328A	5'-GCTGAGCCCCCCATACTCGCTTCCGAGTATGATCCTACC-3'	5'-GGTAGGATCATACTCGGAAGCGAGTATGGGGGGCTCAGC-3'
P406A	5'-GTGAAGCTACTGTTTGCTGCTAACTTGCTCTTGGACAGG-3'	5'-CCTGTCCAAGAGCAAGTTAGCAGCAAACAGTAGCTTCAC-3'
N407A	5'-AAGCTACTGTTTGCTCCTGCCTTGCTCTTGGACAGGAAC-3'	5'-GTTCCTGTCCAAGAGCAAGGCAGGAGCAAACAGTAGCTT-3'
L409A	5'-CTGTTTGCTCCTAACTTGGCCTTGGACAGGAACCAGGGA-3'	5'-TCCCTGGTTCCTGTCCAAGGCCAAGTTAGGAGCAAACAG-3'
W272F	5'-GAGCTGCCAACCTTTTCCCAAGCCCGCTCATG-3'	5'-CATGAGCGGGCTTGGGAAAAGGTTGGCAGCTC-3'
W360F	5'-GGTTCACATGATCAACTTCGCGAAGAGGGTGCCAG-3'	5'-CTGGCACCCTCTTCGCGAAGTTGATCATGTGAACC-3'
W383F	5'-CACCTTCTAGAATGTGCCTTCCTAGAGATCCTGATGATTG-3'	5'-CAATCATCAGGATCTCTAGGAAGGCACATTCTAGAAGGTG-3'
W393F	5'-GATGATTGGTCTCGTCTTCCGCTCCATGGAGCACC-3'	5'-GGTGCTCCATGGAGCGGAAGACGAGACCAATCATC-3'
hERα (LBD, 303-ter)	5'-CATGAATTCAAGAACAGCCTGGCCTTGTCC-3'	5'-CATGCTAGCTCAGACCGTGGCAGGGAAACCCTC-3'

Supplementary Table 3. SEC-SAXS data collection parameters and modeling details

**Data collection parameters**	
Experiment date	August 10, 2014
Beamline/Instrument	APS/BioCAT-18-ID, MAR 165 CCD
Wavelength	1.03 Å
Beam geometry	3 μm (V) x 5 μm (H)
Photon flux	1.3×10^{12} phs/s
Wavelength	1.03 Å
Sample detector distance	3 m
q range	0.005-0.3 $Å^{-1}$
Exposure time	1.1 sec
SEC-SAXS column	Superdex 200 GL 10/300
SEC-elution volume	0.5 µL
SEC flow-rate	0.5 ml/min
Sample injection concentration	3.5 mg/ml
Temperature	10 °C
Software for data reduction	Fit2D[1], ATSAS 2.8.4[2]
Software for SAXS computing	Fast-SAXS-pro[3] and Crysol[4]
Software for structural modeling	In-house iSPOT[5,6]
**Structural parameters**	
R_g from Guinier	38.0 ± 0.3 Å
I(0) from Guinier	4.10 ± 0.04
Molecular mass from *I(0)*	99.7 kDa
Molecular mass from PDB	98.3 kDa
q-range for Guinier fitting	0.017-0.033
q R_g (max)	1.24
Porod Volume	166828 ($Å^3$)
**SASBDB ID**	SASDDU8 (https://www.sasbdb.org/data/SASDDU8)

Supplementary References

1 Hammersley, A. P., Svensson, S. O., Hanfland, M., Fitch, A. N. & Hausermann, D. Two-dimensional detector software: From real detector to idealised image or two-theta scan. *High Pressure Res* **14**, 235-248 (1996).

2 Franke, D. *et al.* ATSAS 2.8: a comprehensive data analysis suite for small-angle scattering from macromolecular solutions. *J Appl Crystallogr* **50**, 1212-1225 (2017).

3 Ravikumar, K. M., Huang, W. & Yang, S. Fast-SAXS-pro: a unified approach to computing SAXS profiles of DNA, RNA, protein, and their complexes. *J Chem Phys* **138**, 024112 (2013).

4 Svergun, D., Barberato, C. & Koch, M. H. J. CRYSOL - A program to evaluate x-ray solution scattering of biological macromolecules from atomic coordinates. *J Appl Crystallogr* **28**, 768-773 (1995).

5 Huang, W., Ravikumar, K. M., Parisien, M. & Yang, S. Theoretical modeling of multiprotein complexes by iSPOT: Integration of small-angle X-ray scattering, hydroxyl radical footprinting, and computational docking. *J Struct Biol* **196**, 340-349 (2016).
6 Hsieh, A., Lu, L., Chance, M. R. & Yang, S. A Practical Guide to iSPOT Modeling: An Integrative Structural Biology Platform. *Adv Exp Med Biol* **1009**, 229-238 (2017).
